# Distributed Architecture for an Integrated Development Environment, Large Trace Analysis, and Visualization

**DOI:** 10.3390/s21165560

**Published:** 2021-08-18

**Authors:** Yonni Chen Kuang Piao, Naser Ezzati-jivan, Michel R. Dagenais

**Affiliations:** 1 Computer Engineering and Software Engineering Department, Ecole Polytechnique Montreal, Montreal, QC h3t 1j4, Canada; yonni.chen-kuang-piao@polymtl.ca (Y.C.K.P.); michel.dagenais@polymtl.ca (M.R.D.); 2 Computer Science Department, Brock University, St. Catharines, ON l22 3a1, Canada

**Keywords:** trace analysis, trace visualization, modular IDE

## Abstract

Integrated development environments (IDEs) provide many useful tools such as a code editor, a compiler, and a debugger for creating software. These tools are highly sophisticated, and their development requires a significant effort. Traditionally, an IDE supports different programming languages via plugins that are not usually reusable in other IDEs. Given the high complexity and constant evolution of popular programming languages, such as C++ and even Java, the effort to update those plugins has become unbearable. Thus, recent work aims to modularize IDEs and reuse the existing parser implementation directly in compilers. However, when IDE debugging tools are insufficient at detecting performance defects in large and multithreaded systems, developers must use tracing and trace visualization tools in their software development process. Those tools are often standalone applications and do not interoperate with the new modular IDEs, thus losing the power and the benefits of many features provided by the IDE. The structure and use cases of tracing tools, with the potentially massive execution traces, significantly differ from the other tools in IDEs. Thus, it is a considerable challenge, one which has not been addressed previously, to integrate them into the new modular IDEs. In this paper, we propose an efficient modular client–server architecture for trace analysis and visualization that solves those problems. The proposed architecture is well suited for performance analysis on Internet of Things (IoT) devices, where resource limitations often prohibit data collection, processing, and visualization all on the same device. The experimental evaluation demonstrated that our proposed flexible and reusable solution is scalable and has a small acceptable performance overhead compared to the standalone approach.

## 1. Introduction

Creating software requires a set of development tools, such as a code editor, a compiler, a debugger, and a profiler, which are often provided by an integrated development environment (IDE). Some popular programming languages such as C++ or Java evolve rapidly and have a very complex syntax. Thus, developing a high-quality compiler or debugger requires significant effort. Traditionally, modern IDEs support multiple programming languages via plugins, but those plugins are tightly coupled to the IDE and hardly reusable in other IDEs [1]. This means that if *m* IDEs support *n* different languages, m×n plugins must be implemented. In recent times, when there are new language versions, updating C++ and Java plugins for widely used IDEs such as Eclipse CDT, in addition to updating the compilers, has become an unbearable effort. Recent work aims to change the traditional plugin approach into a language server approach, thus reducing the number of different implementations to m+n [1]. A language server can be considered as an independent module that provides support for code completion, code errors, syntax highlighting, and other IDE services, through a standard protocol. Moreover, the parser implementation in the language compiler is directly reused as a language server.

However, when those development tools are not sufficient to detect performance defects on large multithreaded systems, developers use tracing techniques to collect data during the software execution. The data collected are eventually written to disk into a trace file, and its size can range from a few megabytes to hundreds of gigabytes. This means that analyzing the trace manually is almost impossible and automated trace visualization tools are required. As the software grows and becomes more complex, it becomes much more efficient for the trace visualization tools to be a part of the IDE, similar to the debugger, in order to effectively and properly collect the required data. For example, Trace Compass is an Eclipse-based trace visualization tool that integrates well with the Eclipse CDT IDE. Among other features, when looking at events in a trace, the source code location of the tracepoint emitting the event can be shown in the IDE.

The current trend towards modular IDEs and language servers is well established [2,3]. However, the use cases for tracing are significantly different from other IDE tools. Indeed, editors, compilers, and even debuggers are centered around the well-defined and structured software package architecture (functions, files, libraries, applications, etc.). In tracing, event streams from several concurrent executions (parallel threads, interrupts, signals, user, and kernel space execution) are multiplexed and can create large trace files of possibly hundreds of gigabytes. Due to this, the modularization of the tracing tools in an IDE presents significant challenges, regarding data communication and where to perform the computation. These issues arise due to the sheer volume of data combined with the various tools and methods, which are not all designed to work together, and while they are certainly manageable issues, there have been no solutions made to address this problem until now.

The recent evolution in software systems also introduces many desirable features or requirements for a new trace analysis architecture. The sources of tracing data are becoming numerous (user space, kernel space, hardware-supported tracing, General-Purpose Graphics Processing Unit (GPGPU), etc.), and each has specific formats, use cases, analyses, and other particular features [4]. Most of the time, the developer then needs to install several different tools that do not interoperate or combine their information. In addition, trace analysis tools may operate in different contexts: an interactive graphical user interface to investigate a local trace file, the same interface to investigate remote trace files from several interacting cloud nodes, an automated anomaly detection module using machine learning to monitor online traces from several cloud nodes, or even a continuous integration framework using trace files to verify the performance of new versions of a software package. Additionally, the growth of the Internet of Things (IoT) has made distributed architectures for trace collection, analysis, and visualization more desirable. Connecting several smart devices and having them interact remotely greatly improves efficiency and organization, yet even when using lightweight monitoring solutions, smart devices often have limited resources, which makes trace data processing directly on the smart device implausible. Furthermore, trace analysis and visualization tools may not be compatible with a given smart device’s hardware [5]. These are requirements that must be considered going forward.

These new requirements motivated us to change how we think about software runtime analysis and to propose a new scalable and efficient IDE-integrated architecture for runtime software tracing, visualization, and analysis. We propose a client–server architecture that includes a backend server for trace collection and analysis, an IDE pluggable client to show the analysis results and to correlate with the source code, and a neutral data transmission protocol between the server and client to ensure loose coupling (Trace Analysis Server Protocol). With this architecture, a monitored device only needs a tracing agent installed, thus making it suitable for resource-constrained IoT devices. Thus, our main contributions, to address the current situation, where trace visualization tools do not support several important new use cases and cannot integrate well with the new modular IDEs, are the following:

1. A new scalable and efficient modular client–server architecture for large trace analysis;
2. The Trace Analysis Server Protocol, collaboratively developed with the EffiOS team and the Ericsson team, alongside our team;
3. A prototype implementation of this proposed architecture;
4. A quantitative evaluation of the scalability, efficiency, and overhead of the proposed implementation.

To our knowledge, this is the first trace visualization tool that addresses the challenges brought forward by the recent modularization of IDEs, with a frontend user interface and backend servers for language parsing, debugging, and now tracing. The Trace Analysis Server Protocol is the critical part of this proposed architecture, which allows development tools to interoperate in any IDE that implements the protocol. The evaluation of our work and its success will be based on a number of factors, including the assurance of a low overhead, as well as the scalability of the proposed architecture. We consider the overhead low if it remains less than 100 ms, which is the point where a delay would become perceptible to a user [6]. While the primary contribution of this paper is the architecture, its evaluation is focused on the implementation of the architecture and its capabilities.

This paper is organized as follows. First, we review the related work and the existing trace visualization tools in Section 2. Then, we present the specification of the proposed architecture and detail its implementation in Section 3. In Section 4, we evaluate the proposed solution by measuring the data transferred, the performance overhead, and the scalability. Finally, we conclude and present possible future work.

## 2. Related Work

The related work is divided into three sections. The first section reports recent works on IDEs. The second section covers tracing and trace analysis from a high-level perspective. The third presents existing trace visualization tools and their architecture.

### 2.1. Recent Works on IDEs

Microsoft’s Language Server Protocol (LSP) is a communication protocol, based on JSON-RPC, between a client, which is the IDE, and a server that offers language support [1]. At this time, the LSP does not specify how the messages exchanged should be transferred and the client is responsible for managing the server’s lifetime. The protocol provides common features such as code completion, code error, syntax highlighting, and go-to definition. Many organizations such as the Eclipse Foundation, GitHub, and JetBrains are adapting their popular IDEs (Eclipse, Atom, IntelliJ) to implement the LSP.

Keidel et al. presented Monto [1], which follows the same idea as the LSP, but their approach allows the language server to be stateless. Services are responsible for providing the common language features, and those services may also be composed of smaller services. Moreover, in comparison to the LSP, their solution does not have to maintain and update a copy of the source code.

Marr et al. presented the Kómpos protocol [7], which is a concurrency-agnostic debugger protocol. Its goal is to decouple the debugger from the concurrency models such as threads, locks, communication event loops, and others. The protocol provides support for common features such as breakpoints, step-by-step, and visualization of the interaction of concurrent models. In comparison to the existing debugger protocol such as the Java Debug Wire Protocol or the GDB machine interface, their solution is not specific to a concurrency concept.

Efftinge et al. presented Theia [2], a new open-source IDE framework for building IDEs that could run both as a desktop application or in a web browser connected to a remote backend. The project shares many similarities with Microsoft’s code editor, Visual Studio Code. Theia uses Node.js and Electron and implements the LSP.

### 2.2. Tracing and Trace Analysis

Tracing is a technique that can be used to aid in performance analysis, which records runtime events on an executing program. Before collecting those events, tracepoints must be inserted either statically or dynamically. In the first case, the code must be modified to include tracing macros and must be recompiled. In the second case, tracepoints are added dynamically to a compiled and running program [8]. At runtime, events will be emitted, and a program called tracer will capture them to eventually produce a trace file, organized in a specific format. Tracing can be performed on several levels including the user space, kernel, hardware, hypervisor, network, etc. Given that tracing is used to detect and identify performance issues, a tracer must have a minimal execution overhead. A trace analysis technique is used to transform the trace events into states and reorganizes them into a tree structure, for faster access [9]. Indeed, trace files could easily contain millions, even billions, of events, and the analysis must use an efficient data structure to maintain query performance.

Existing works have established execution trace analysis as an effective tool for IoT device monitoring, particularly for the purposes of improving security. HADES-IoT [10] uses kernel tracing as a lightweight solution for high-accuracy intruder detection. Gassais et al. [5] presented an improved host-based intrusion detection system for the IoT where an analysis system is separate from the tracing agents installed on smart devices. This framework is capable of high precision while introducing very little overhead on each IoT device.

### 2.3. Trace Visualization Tools

There are many standalone tracing tools that support different solutions of tracing and trace analysis. Ezzati-Jivan et al. presented most of them in a survey [11]. However, in this section, we only review the recent open-source tools that can support multiple trace formats, large traces, and user-defined analysis and some communications.

LTTng Scope [12] is a JavaFX-based desktop trace viewer that focus only on the Common Trace Format (CTF) [13] traces produced by the LTTng tracer [14]. The architecture is well defined, separating the trace analysis in a reusable library, called Jabberwocky, and the visualization part in another component. The trace analysis library supports large traces and exposes an API that the visualization component invokes directly. However, LTTng Scope does not support user-defined analysis and other trace formats.

TraceCompass is an Eclipse-based desktop trace visualization tool. It supports different trace formats and offers many comprehensive analyses [9]. In their previous work, Kouamé et al. already studied and implemented an XML-based language for user-defined analysis in TraceCompass [15]. This tool supports large traces, but its architecture is monolithic and not well defined; some components from the views are tightly coupled to components from the analysis, which can result in them being difficult to maintain.

Google has its tracing infrastructure system within Chromium [9]. To view the trace analysis results, a JavaScript frontend, called Trace-Viewer, has been developed. It supports the Trace Event Format [16] produced by Chrome Tracing and Android Systrace, but can also write a trace format importer.Tracing, trace analysis, and visualization are separated into different architectural layers. However, it does not have the features required to support user-defined analysis.

OpenZipkin [17] is a distributed tracing system used to trace microservices’ architecture-based systems created by Twitter. It is a tracer implementation of the OpenTracing specification. The architecture is organized as a client–server architecture; the backend is written in Java and uses Spring Boot, while the client is written in JavaScript. The client and the server communicate through a custom HTTP Representational state transfer (REST) API. This tool is limited to OpenTracing and does not support other trace formats. Moreover, it does not support large traces and user-defined analysis.

Jaeger [18] is another tracer implementation of the OpenTracing specification by Uber Technologies. The architecture is organized as a client–server architecture where the server is written in Go and the client is written in JavaScript. The client and the server communicate through a custom HTTP REST API. As OpenZipkin, this tool does not support other trace formats, large traces, and user-defined analysis.

## 3. Proposed Solution

**Figure 1:**
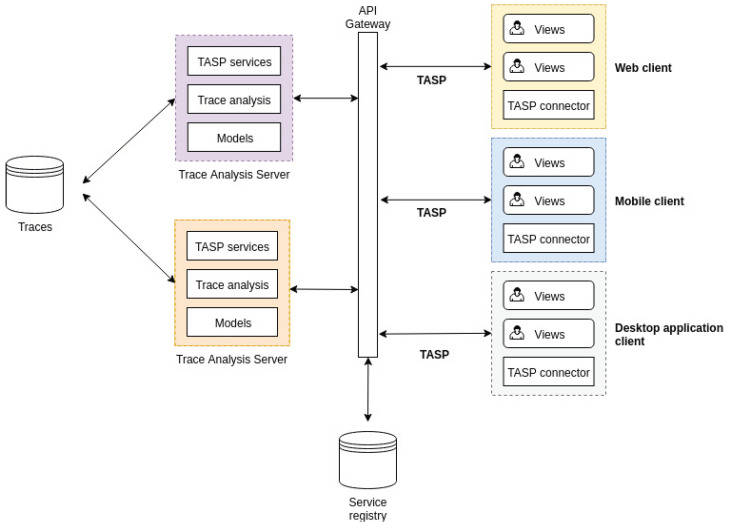
Client–server architecture for distributed trace analysis.

Our proposed solution consists of a client–server architecture with multiple layers that have different roles and responsibilities. Figure 1 represents an overview of the proposed architecture and its components, which will be explained in detail in the following sections.

The architecture separates traces, trace analysis, and clients. By applying the separation of concerns principles, each component of the architecture is independently maintainable. On the left, we have the element responsible for storing and managing traces that can be of different sizes and in various formats (e.g., CTF, Trace Event Format). Then, we have the trace analysis server, responsible for providing specific analysis results on any input trace. Each server may offer different analyses or trace format support, so we need a mechanism to dispatch client requests to the right server. The API gateway pattern solves this problem [19]. When we add or remove servers, we need a mechanism to update which analysis or feature is available, so we use the server-side service discovery pattern [20] to this end. Finally, we have the clients, responsible for consuming and showing the data produced by the server in useful views to the user. A client can be any system interested in showing trace analysis results whether it is an IDE, a monitoring system, or a continuous integration system.

To support heterogeneous clients, we introduced the Trace Analysis Server Protocol (TASP), which requires TASP connectors in the clients and TASP services in the trace analysis server. We assumed the heterogeneous nature of the clients because they may have different amounts of computing power and may be written in different programming languages. The communication via the TASP is performed over the network, which allows the trace analysis server deployment to be in a cluster of high-performance computers. Therefore, our proposed architecture could profit from distributed systems, benefiting scalable computing power and resources while also having the trace analysis results available anywhere at any time without depending on a specific program installation to view them. If the deployment in the cloud is not the desired scenario, our solution can also be deployed locally.

Even though the server performs the whole calculation workload, communication between the client and the server through the TASP could be expensive. Indeed, compared to a monolithic architecture, there is an execution time overhead related to setting up the connection, the network latency, and the serialization/deserialization operations. The client also has an essential role in the architecture to reduce the number of requests to the server. For this reason, we also detail the architecture on the client-side by presenting its components, the caching mechanisms, and other techniques designed to this end.

### 3.1. The Trace Analysis Server Architecture

**Figure 2:**
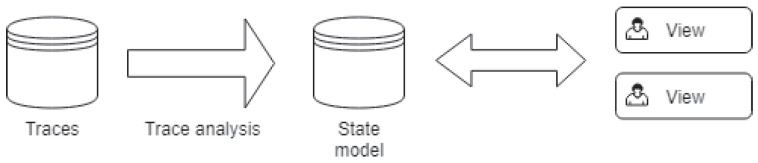
Common trace analysis approach.

For the determination of the architecture to be used for our solution, we first take a look at existing architectures and decide on one based on those. Wininger et al. [21] explained, in their previous work, illustrated by Figure 2, the common trace analysis approach. A trace analysis reads a trace sequentially and stores its results (called state models) in a data structure that can be written on disk. From the trace events, the analysis would extract the intervals of the state values for different system resources (CPU, processes, disks, etc.) [22]. Montplaisir et al. [22] already studied and proposed an efficient model to query such a data structure. Then, views query this data structure, filter the relevant information, and transform them into a high-level model to be displayed on charts.

**Figure 3:**
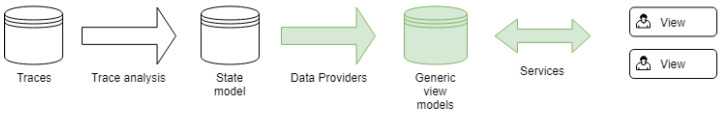
Proposed trace analysis server architecture.

Any system interested in showing the analysis results would use a library that queries the state models. However, this approach has two limitations. First, if the involved systems are heterogeneous, the library must serialize the state model into an interoperable format such as the Extensible Markup Language (XML) or the JavaScript Object Notation (JSON). Prieur et al. [23] showed that the data structure containing the state model can be massive (reaching gigabytes) depending on the trace. Therefore, serializing the state models directly is not efficient nor suitable if the data are transferred through the network. Second, the results of a query to the data structure containing the state models are not directly practicable, and the visualization systems must transform them. This means that every different system interested in showing a particular trace analysis result must implement its transformation of the state models before displaying it to the user. Those limitations motivated us to introduce new architectural components, shown in green in Figure 3, in the trace analysis process. This is the architecture used for our solution.

To have the smallest possible data serialization and avoid rewriting code to transform the state models, we chose to utilize a Service-Oriented Architecture (SOA) approach. Our proposed trace analysis server architecture introduces the Data Provider component, responsible for querying the state models’ data structure, filtering the results, and transforming them into generic high-level models. Any system interested in showing the generic models queries the Service component via the TASP. The Service component is responsible for implementing the TASP and serializing the model computed by the Data Provider component. The model received by those systems is practicable, and there is almost no modification needed to display it directly in charts.

Applying SOA principles in the development of a system facilitates its scalability, evolvability, manageability, and interoperability [24]. In the context of trace analysis, those qualities are necessary for supporting the addition or modification of new features, such as user-defined analysis, while reducing the cost of developing them. For those reasons, we chose an SOA approach for the trace analysis server, and its composition is detailed below. Before deciding how clients would query the Service and the Data Provider components, we must determine what they are querying.

#### 3.1.1. Extracting Generic View Models

**Figure 4:**
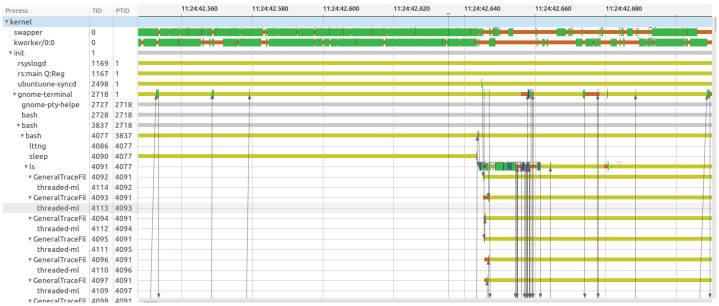
The thread status view in TraceCompass is an example of a time graph view.

The trace visualization tools presented earlier provide many different useful views for displaying the analysis results. One of the most important types of view is time graphs. Figure 4 shows an example of a time graph view in TraceCompass. Other trace visualization tools such as Trace-Viewer or LTTng Scope provide the same type of view, but within a different user interface.

On the left side of the view, we see a list of entries organized in a tree hierarchy. In practice, those entries are routines such as a process, a thread, or a function that have a start time, an end time, and a name. All of the time values could be represented as the Unix Epoch time in nanoseconds. On the right side of the view, we see a Gantt-like chart that shows rectangles of different colors, which we call states. These states are bounded by a start time and an end time. Each time graph routine on the left is associated with a collection of states on the right, which we call a row. Each state has a value, which the view maps to a rectangle color, and may also have a label. Along with the Gantt-like chart, we may have arrows, starting from a state of a routine to another state of another routine. Those elements are common to all trace visualization tools presented. In practice, time graph views are used to show the results of the critical path analysis, the flame graph analysis, or the call stack analysis. From the previous elements, we extracted common models for time graphs views, which are displayed here as Listing 1:

**Listing 1:** Code of Time graph models definition.

typedef long timegraph_routine_id;

struct TimegraphRoutine {
timegraph_routine_id id;
timegraph_routine_id parentId;
long startTime;
long endTime;
string name;
}

struct TimegraphState {
long startTime;
long endTime;
int value;
string label;
}

struct TimegraphRow {
timegraph_routine_id routineId;
TimegraphState[] states;
}

struct TimegraphArrow {
timegraph_routine_id sourceId;
timegraph_routine_id destinationId;
long startTime;
long endTime;
}

The second important type of view that we focused on is the XY views. An XY view displays on a chart one or multiple series, which is merely a mapping between a collection of *x* and *y* values. Each series on the chart has a name. Those views can be shown as a bar chart, as a line chart, or as a scatter chart. In practice, XY views are used to display the results over time of the CPU usage analysis, the memory usage analysis, the disk I/O activity analysis, or the counter analysis. As an additional feature, Trace Compass also displays on the left side of the view a list of selectable entries used for filtering series. For example, the CPU usage view in Trace Compass displays processes and threads on the left, allowing the user to filter the CPU usage per process. The filtered series then appear on the right side so that they can be easily compared. Considering that, Listing 2’s extracted model is shown here:

**Listing 2:** Code of XY models’ definition.

struct XYSeries {
long id;
string name;
long[] x;
double[] y;
}

struct XYModel {
string name;
XYSeries[] series;
}

These models were designed to be generic and compact. By being generic, they can be created either by LTTng Scope, Trace-Viewer, or Trace Compass. Moreover, the model applies to any new trace analysis implementation, the results of which can be displayed either in a time graph view or an XY view. By being compact, only the smallest relevant information is serialized and transferred over the network to the client. Finally, the introduced models have no direct links to the state models, which decouples the trace analysis internal implementation from the clients.

#### 3.1.2. The Data Provider Layer

**Figure 5:**
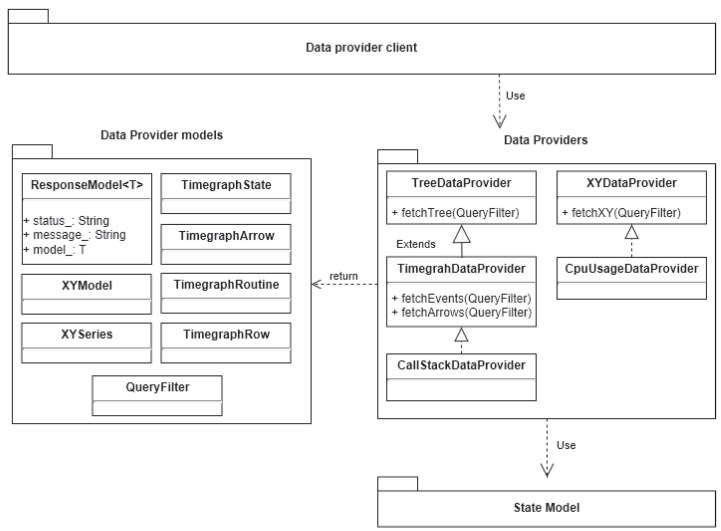
Class diagram of the Data Provider layer.

This layer of abstraction is used as an interface to build and fetch the generic models presented earlier. Figure 5 shows the class diagram of the Data Provider layer. Therefore, we have different interfaces for each model: one for fetching tree-like models, one for fetching XY models, and one for fetching time graph models. The implementation of any of these interfaces is a class that gives the results of an analysis.

The Data Provider interfaces also provide a mechanism for fetching a specific model given a QueryFilter defined below. In practice, we are interested in models bounded in a particular interval of time; when the user zooms in or out or pans left or right, the bounds in the QueryFilter must change accordingly. Furthermore, depending on the resolution of the computer screen, we may be interested in showing a lower or higher definition of the model. Finally, we may be interested in particular data entries that have an id such as the TimegraphRoutine or the XYSeries. Thus, the Data Provider must allow fetching only them. This mechanism gives much flexibility to the Data Provider client, but the primary motivation is to minimize the amount of data to transfer and the serialization overhead. If the client is only interested in querying a specific part of the model, there is no need to return the results for the whole analysis. However, it is still the client’s responsibility to optimize queries to the data provider. Listing 3 shows us the definition for the QueryFilter:

**Listing 3:** Code of QueryFilter definition.

struct QueryFilter {
long start;
long end;
int count;
long[] selectedIds;
}

The Data Provider layer is also designed to support nonblocking fetch operations. Indeed, for large traces, the analysis could be very time-consuming. For this reason, the fetch method returns a ModelResponse that encapsulates a model, a status, and a status message. The status is simply an enum, mapping integers with a state: running, completed, failed, or canceled. When we obtained a failed status, we may be interested to know why. That is why the ModelResponse has a status message, a string detailing the status state.

For example, assume that a Data Provider client is interested in a time graph model from $t_{1}$ to $t_{10}$. When receiving the request, the data provider may not have the analysis result up to $t_{10}$, but up to $t_{5}$ only. Instead of waiting for the analysis to complete, the data provider will return a ModelResponse with running as the status and the model associated with $\left[t_{1},t_{5}\right]$. It is the client’s responsibility to request the data provider as long as a completed status is not received.

#### 3.1.3. The Service Layer

While the Data Provider layer provides a simple API for fetching models, the service layer is responsible for serializing them and for providing interoperability with the clients. This layer can be considered as a facade of the trace server analysis that implements the TASP. To ensure interoperability, many mechanisms exist such as remote procedure calls (RPC) or object request brokering (e.g., CORBA) [25]. The trace analysis server and the client must use the same mechanism, so the chosen approach must be implemented in different programming languages. Finally, the Service layer is highly coupled to the TASP specification, which means that the service implementation is influenced by the network protocol used for the TASP.

### 3.2. Trace Analysis Server Protocol

**Figure 6:**
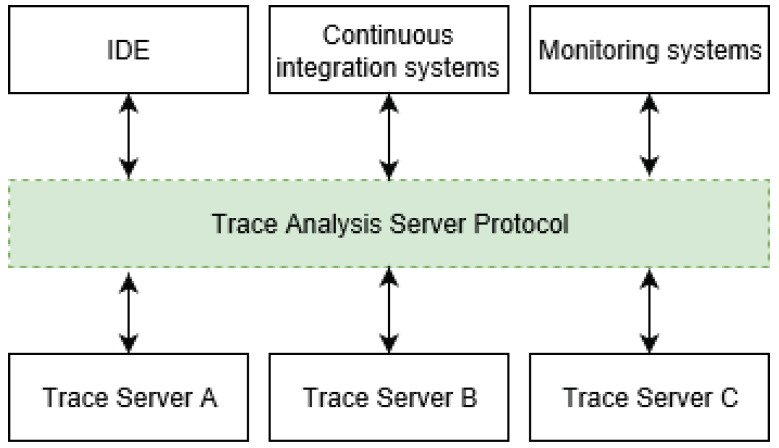
Interaction relying on the Trace Server Analysis Protocol.

The Trace Analysis Server Protocol (TASP) is profoundly influenced by the Language Server Protocol (LSP). As shown in Figure 6, its goal is to standardize how multiple development tools or clients communicate with trace analysis servers. Thus, any IDE that implements the TASP could interoperate with a trace analysis server and shows the same views provided by the standalone trace visualization tools. Moreover, the TASP makes trace servers interchangeable, and their implementation is abstracted for the client.

However, the LSP relies on the JSON-RPC protocol [26], while we chose an HTTP Representational State Transfer (REST) approach. Both the JSON-RPC and HTTP protocols are stateless and implemented in the most popular languages, but they do not provide the same flexibility in the serialization format. JSON-RPC uses the JSON format only, which could be suboptimal regarding the data size [27]. Serializing the models into a text-based format could easily reach megabytes, and the TASP should serialize them into a binary-based format to minimize the data transferred. Using compression (GZip) over JSON could potentially reduce the size of the data, but this approach implies using more CPU resources and leads to more execution time overhead. HTTP provides a mechanism to send binary data by setting the HTTP response header Content-Type to application/octet-stream. With this in mind, Google’s Protocol Buffers, also called Protobuf [28], was chosen as the binary format to serialize the models. We determined that using a binary format such as Protobuf was more efficient than even a compressed JSON format. We explore these serialization formats more in depth in Section 4, where we compare them with each other to justify our decision to use a binary format.

Masse et al. presented a guide for designing consistent RESTful APIs [29] that we followed for the specification of the TASP. Its full specification is available on Theia IDE’s GitHub page [30]. The TASP design is a result of collaborative work between the EffiOS’ team working on LTTng Scope, the Ericsson’s team working on TraceCompass, and our research lab. The goal of this collaborative work was to propose a protocol that would be the least specific to the inner workings of a particular trace analysis tool, making it interoperable.

### 3.3. The Client Architecture

The proposed client architecture has three principal components: TASP Connector, Widget, and Chart. The TASP connector is responsible for querying the server and for returning a view model entity. The Chart obtains a view model and is responsible for drawing it and displaying it. Finally, the Widget acts as a controller. Because the TASP Connector and the Chart do not know each other, the Widget is responsible for calling the TASP Connector’s methods and feeding the returned model to the Chart. As we explained, communication between the TASP Connector of the client and the TASP services of the trace analysis server could be expensive, and the Widget component has an important role in reducing the requests sent to the server. To transfer as few data as possible, the client is also responsible for narrowing its request to the minimum. In this section, we cover how the client manages the amount of data it needs to be transferred from the server. We explain how the client can optimize its request to limit the data transferred, and we present two techniques to reduce the number of requests to the trace analysis server: throttling and caching.

#### 3.3.1. Reducing the Size of Transferred Data

The client should have information about the size or resolution of the screen being used. Let us say that the client wants to display an XY view on a screen with a 1920×1080 pixel resolution. The worst case is that the view takes the whole screen, which means that we need to query one point per horizontal pixel. Thus, we do not need to query more points than the number of available pixels. The same idea goes for the time graph states and entries. We may have thousands of entries, but we may not have enough vertical pixels to show them. The given example was for a standard 1080p screen, but the client must be able to adapt to larger screen resolutions such as 2 K or 4 K.

#### 3.3.2. Throttling

Throttling is a technique for controlling the consumption of resources by an application [31]. As we explained above, the client might receive an incomplete model when querying the server. As long as the completed status is not received, the TASP connector may make *N* consecutive requests to the server. For example, suppose that 10 s are needed to obtain the complete view model from $t_{1}$ to $t_{100}$, each request transfers 100 KB, and each request and redraw operation takes 200 ms. This means that when the user interface is refreshed five times per second, we need to make 50 requests to the server until completion and we transfer 5 MB. Throttling helps us reduce this number if we wait a certain time *x* before retriggering the request and redraw operations. Let us say that we chose to wait for 800 ms; this means that we will need ten requests to the server instead of 50. Consequently, the user interface is refreshed only once per second, and we transfer five times fewer data (1 MB instead of 5 MB). The longer the waiting time, the less requests we make to the server. However, the drawback of this approach is that the user interface becomes less responsive.

#### 3.3.3. Caching

The idea of this technique is that the client maintains a cache and looks into it before requesting the server. If the client finds the desired information in its cache, it does not need to query the server. This technique is used to limit communication between the client and the server, but it leads to more memory consumption for the client and a mechanism to manage the lifetime of the objects in the cache. In order to save requests, we also save time as looking in a local cache does not have network latency concerns.

**Figure 7:**
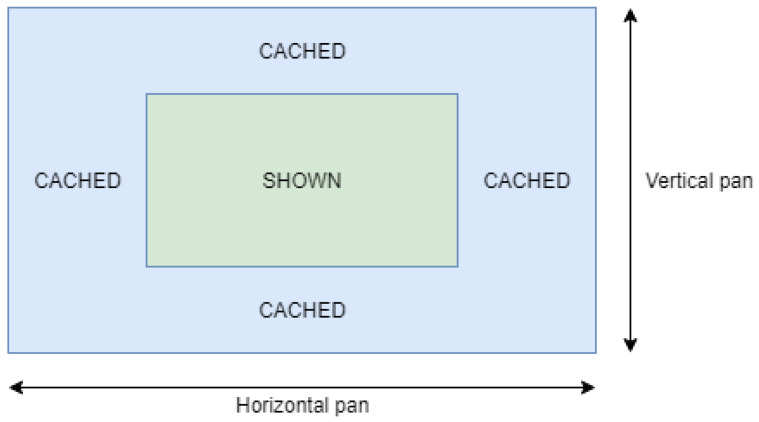
Vertical and horizontal caching for the time graph.

We used caching for the view models. Instead of querying only the view model that is to be displayed on-screen immediately, we query more. For example, let us take the time graph use case. We are interested in showing the time graph row model of t=[100,200] for entries e={3,4,5}. Instead, we query the server for $t^{\prime }=\left[75,225\right]$ and $e^{\prime }=\left\{1,2,3,4,5,6,7\right\}$, but only display the data described by *t* and *e*. As shown in Figure 7, the green component is what the user sees, and we have the blue component in the cache. This approach can be seen as the opposite of the first suggested technique because we do not request the strict minimum, but both approaches complement each other. Indeed, a combination of both techniques leads to better results when the user is panning vertically or horizontally. For example, let us say that instead of requesting 100 KB of data, we query 140 KB because of caching. Every time that the user is panning in a way that what will be shown is available in the cache, we save a request of 100 KB.

However, this technique does not work well when the user is zooming in or out, because every zoom operation leads to a new request. Thus, if the user is mostly zooming, this technique leads to worse results for the total of data transferred. For this reason, we suggest having a reasonable cache size, which is arbitrarily 25–50% larger than the shown area. Additionally, caching is also useful for models that will not or are not likely to change according to the resolution or time interval. Take for example the disk I/O activity analysis. The list of drives is unlikely to vary according to the resolution or the time intervals. Thus, the model can be requested only once and stored in the cache.

## 4. Evaluation

In this section, we focus on evaluating the proposed solution. To do this, we implemented the client–server architecture. While the proposed architecture solves the initial problems regarding modularity and scalability, we still need to ensure, for a wide range of trace sizes, that the amount of data transferred is small and the performance overhead is acceptable in comparison to the standalone trace analysis tool. This evaluation primarily focuses on the implementation of the architecture, rather than the architecture itself, as it is much more tangible to assess an implementation of an architecture.

### 4.1. Implementation

The full implementation of the server architecture is available in the Trace Compass main project and incubator project. The Data Provider implementation is on the master branch of the main project, while the HTTP services implementation is on the master branch of the incubator project:

- https://git.eclipse.org/r/#/q/project:tracecompass/org.eclipse.tracecompass (acce- ssed on 10 February 2021)
- https://git.eclipse.org/r/#/q/project:tracecompass.incubator/org.eclipse.tracecompass.incubator (accessed on 10 February 2021)

We also implemented the client architecture, written in JavaScript and running within a web browser, which queries Trace Compass through the TASP. This implementation, named TraceScape, is available as an open-source project in the following GitHub repository: https://github.com/cheninator/trace-scape (accessed on 10 February 2021).

### 4.2. Tests Environment

All the tests were executed on localhost on a machine with an Intel Core i7-6700 @ 3.40 GHz, 16 GB RAM, and running Ubuntu 16.04 LTS with the kernel 4.13.0-41-generic. The web client uses the official build of Chrome 65, and the Trace Compass server runs on OpenJDK Version 1.8.0_171. A trace of 2.47 GB was generated by tracing all kernel events of the test machine using LTTng 2.10.3. The trace contains 1580 threads and around 75 million events. The generated trace is an ideal candidate since we want to show that our solution supports large traces (size on disk greater than 1 GB). After completion of the thread status analysis and the kernel memory usage analysis, their respective state model size on disk were 1.9 GB and 212 MB. The trace and the state models were stored on a 120 GB SanDisk SSD.

In the tests conducted to measure the data transferred and the execution time overhead, the client sends requests to the server, for the analysis results from the start to the end of the trace, with realistic values for the resolution. Moreover, the client sends requests for different models; XYSeries for the kernel memory usage analysis and TimegraphRow for the thread status analysis. For consistency, we made sure to request the same collection of TimegraphRow and XYSeries.

Three factors can influence the data transferred and the execution time overhead: the desired resolution of the model, the number of different TimegraphRow or XYSeries, and the size of the trace. In each test, we fixed two out of these three factors and varied the other. For each test result, the full-line series were the results of the average of 10 executions, and the dotted line series was the average plus or minus the standard deviation.

### 4.3. Data Transferred

The quantity of data to be transferred during a single session depends greatly on how many requests are made from the client to the server. Instead of evaluating it, we chose to measure the data transferred per request. We compared three serialization formats used: JSON, JSON compressed with GZip, and Protobuf.

#### 4.3.1. XY Models

**Figure 8:**
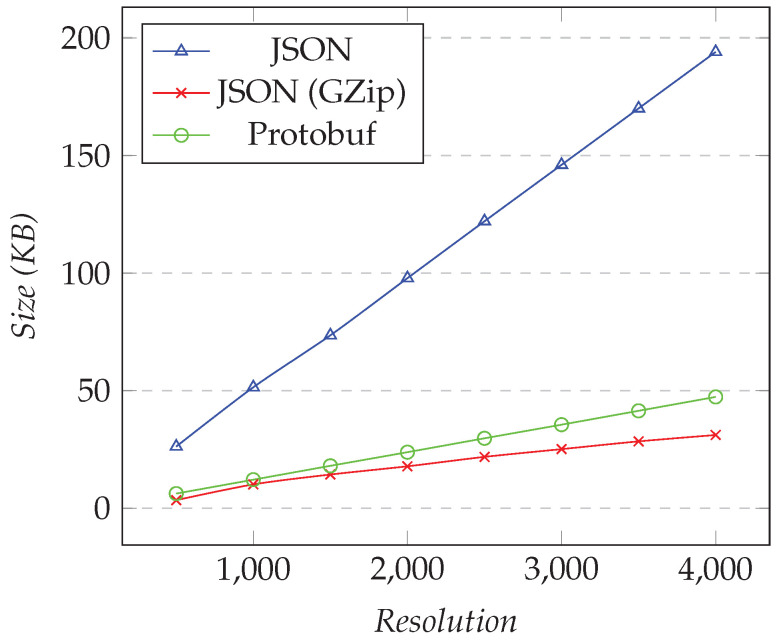
Comparison of the amount of data transferred for requesting the XY model. Fixed number of XY series set to 1, changing the resolution.

**Figure 9:**
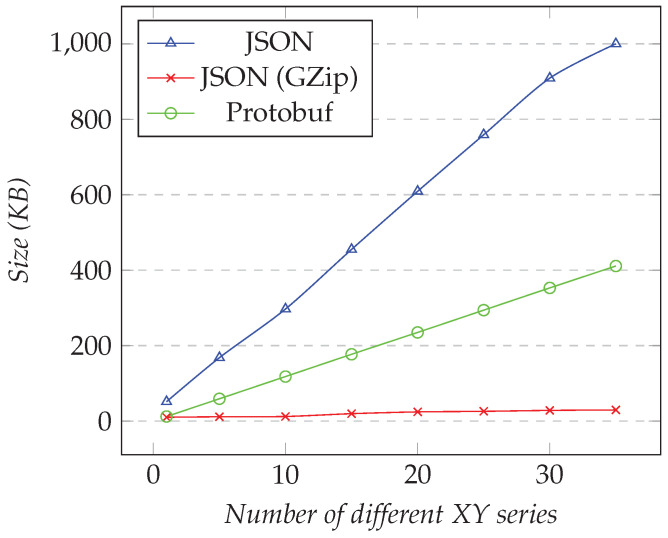
Comparison of the amount of data transferred for requesting the XY model. Fixed resolution set to 1000, changing the number of different XY series.

In the first test, we requested only one XY series, and we changed the desired resolution (i.e., the number of points). Figure 8 shows the results for the first test. In the second test, we fixed the resolution to 1000 and changed the number of different XY series. Figure 9 shows the results for the second test.

As the resolution increased, the amounts of the data transferred over Protobuf and compressed JSON became close. Compared to JSON, using compression gave around 80–87% of space-savings, while Protobuf was more constant, with 75–77% of space-savings. Finally, for 4000 points, which is near a 4 K monitor, we transferred less than 50 KB per request for both Protobuf and compressed JSON, while nearly 200 KB was needed for JSON.

However, as the number of XY series increased, using compression was much more efficient than Protobuf. Figure 9 shows that GZip was better than Protobuf in this test. Compared to JSON, the compression gave around 80–97% of space-savings, while Protobuf gave around 59–76%. Finally, when we requested 35 XY series, we needed to transfer less than 30 KB for compressed JSON, while approximately 420 KB was necessary for Protobuf.

#### 4.3.2. Time Graph Models

**Figure 10:**
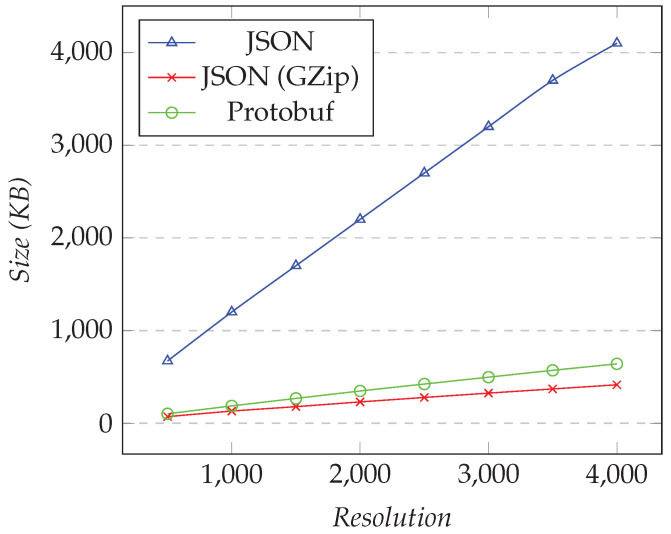
Comparison of the amount of data transferred for requesting the time graph row models. Fixed number of time graph row set to 25, changing the resolution.

**Figure 11:**
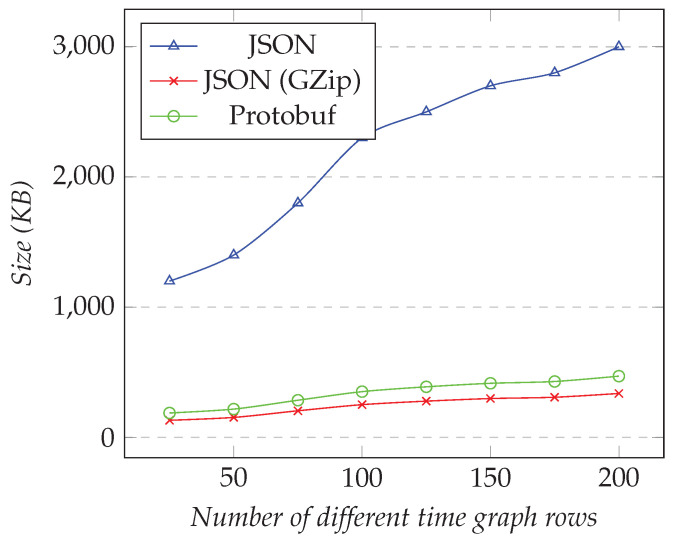
Comparison of the amount of data transferred for requesting the time graph row models. Fixed resolution set to 1000, changing the number of time graph rows.

We performed similar tests for the time graph models. In the first test, we fixed the number of different time graph rows to 25, and we changed the resolution. Figure 10 shows the results for the first test. In the second test, we fixed the resolution to 1000, and we changed the number of different time graph rows. Figure 11 shows the results.

As XY models, as the resolution increased, the amount of data transferred over Protobuf and compressed JSON were close. Compared to JSON, using compression gave around 90% of space-savings, while Protobuf gave around 84% of space-savings. Finally, for a resolution of 4000, we transferred less than 500 KB per request for compressed JSON, nearly 650 KB for Protobuf, and over 4 MB for JSON.

However, unlike XY models, compressed JSON was not significantly more efficient than Protobuf as the number of time graph rows increased. If we compared it to JSON, the space-savings were around 88–89% and approximately 84% respectively for compressed JSON and Protobuf. Finally, when we requested 200 time graph rows, about 500 KB was transferred for Protobuf, less than 350 KB for compressed JSON, and 3 MB for JSON.

### 4.4. Execution Time Overhead

The client–server architecture introduces an execution time overhead. Given that the tests were run on localhost, this overhead was mainly composed of opening/closing TCP connections, sending/receiving HTTP headers, and serializing/deserializing the data. The network latency must be considered when we are not in a localhost environment. The execution time overhead is given by:

(1) $$\begin{array}{c} Overhead\left(ms\right)=T_{TCP}+T_{Headers}+T_{Serialization} \end{array}$$

To obtain the view models, the client makes an HTTP request to the server, which queries the Data Providers. Thus, the time for completing a request from the client to the server is the sum of the time for querying the Data Provider and the execution time overhead:

(2) $$\begin{array}{c} T_{Request}=Overhead\left(ms\right)+T_{Query} \end{array}$$

On the web client, we measured the time for completing a request to the server. On the server, we measured the time for querying the Data Provider. To obtain the execution time overhead, we subtracted the two measured execution times. Aside from having the execution time overhead in milliseconds, we were also interested in obtaining the execution time overhead as a percentage:

(3) $$\begin{array}{c} Overhead\left(\%\right)=\frac{Overhead(ms)}{T_{Query}}*100 \end{array}$$

Given that the serialization time depends significantly on the size of the model, we showed in this test how the execution time overhead varied depending on the model and its size.

#### 4.4.1. XY Models

**Figure 12:**
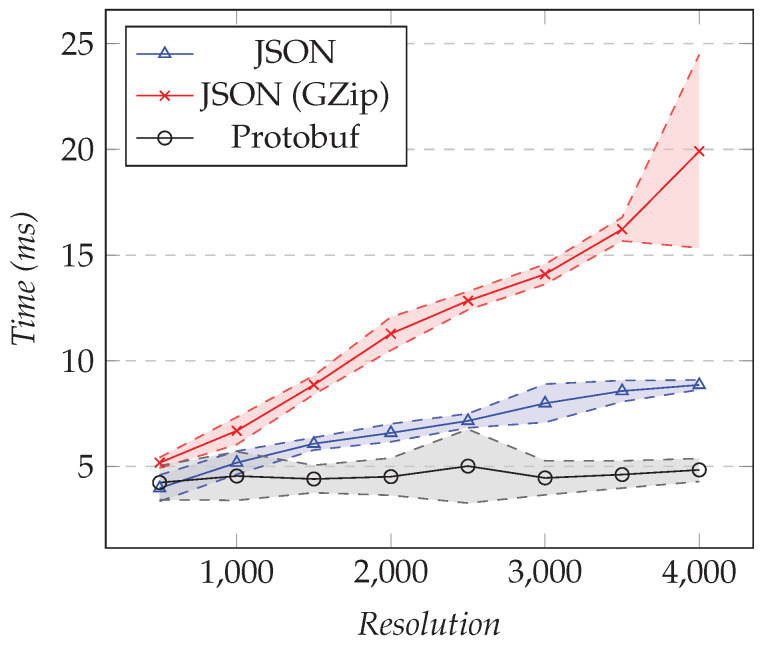
Comparison of the execution time overhead for requesting the XY model. Fixed number of XY series set to 1, changing the resolution.

**Figure 13:**
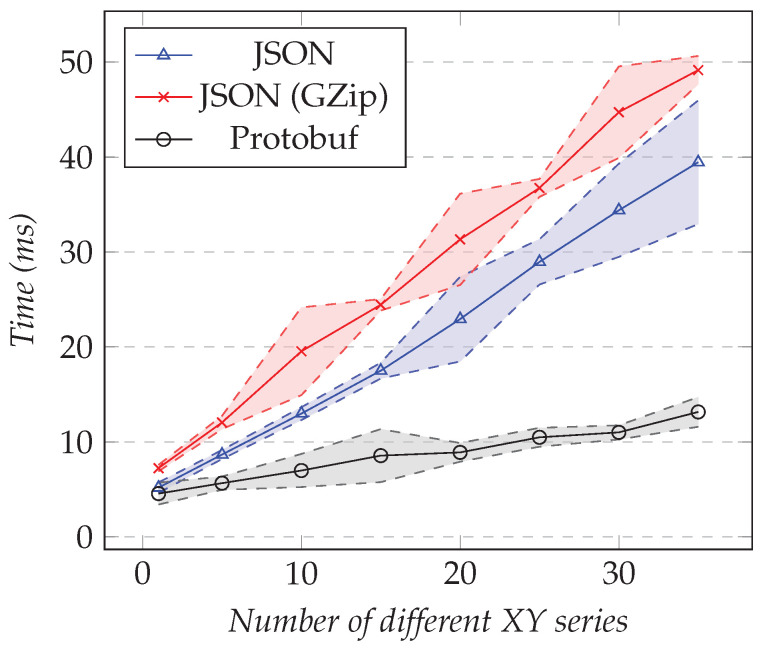
Comparison of the execution time overhead for requesting the XY model. Fixed resolution set to 1000, varying the number of different XY series.

We reused the same test configurations presented in the previous section, but for measuring the execution time overhead instead of the amount of data transferred. Figure 12 shows the results of the first test and Figure 13 the results of the second test.

As the resolution increased, the execution time overhead in milliseconds increased as well, which was expected because the size of the model affected the serialization time. However, the cost for Protobuf seemed fairly constant for the tested resolutions: as seen in Figure 12, the time never went significantly above 5 ms, while the other two were both on an upwards slope. There was indeed a fixed cost for setting up the connection, and thereafter, the serialization with Protobuf was extremely efficient. Unsurprisingly, using compression had more execution time overhead than using JSON. Finally, for 4000 points, there was around 5 ms of overhead for Protobuf, while it reached 20 ms for compressed JSON and 9 ms for JSON. Table 1 shows the overhead as a percentage.

Compressed JSON led to 11.4% overhead for 500 points and 7.3% overhead for 4000 points, while Protobuf led to 9.9% and 1.8%, respectively. In between, we had 7.1% and 3.4% overhead by using JSON directly. Those results showed that when we requested more points, the trace analysis server spent more time to compute the model than to serialize it.

As the number of XY series increased, the execution time overhead in milliseconds increases as well. For 35 XY series, it reached nearly 50 ms for compressed JSON, 40 ms for JSON, and 14 ms for Protobuf. Even if those times seem to be small, the percentage, on the other hand, was high. Table 2 shows the overhead as a percentage.

Using compression led to the highest overhead for the execution time. It reached 7.8% for one XY series and 47.9% for thirty-five. On the other hand, Protobuf had the smallest, leading to 4.8% overhead for one XY series and 13.2% overhead for thirty-five. In between, we had respectively 5.1% and 40.9% overhead by using JSON. Those high percentages are explained by the fact that querying more entries from the Data Provider did not take much more time than serializing the computed result.

#### 4.4.2. Timeline Models

**Figure 14:**
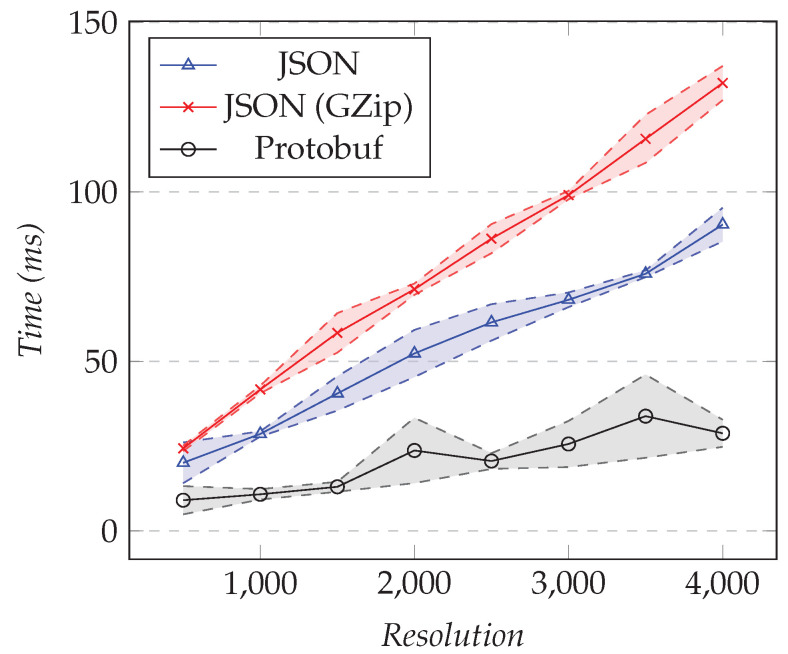
Comparison of the performance overhead for requesting time graph row models. Fixed number of time graph rows set to 25, changing the resolution.

**Figure 15:**
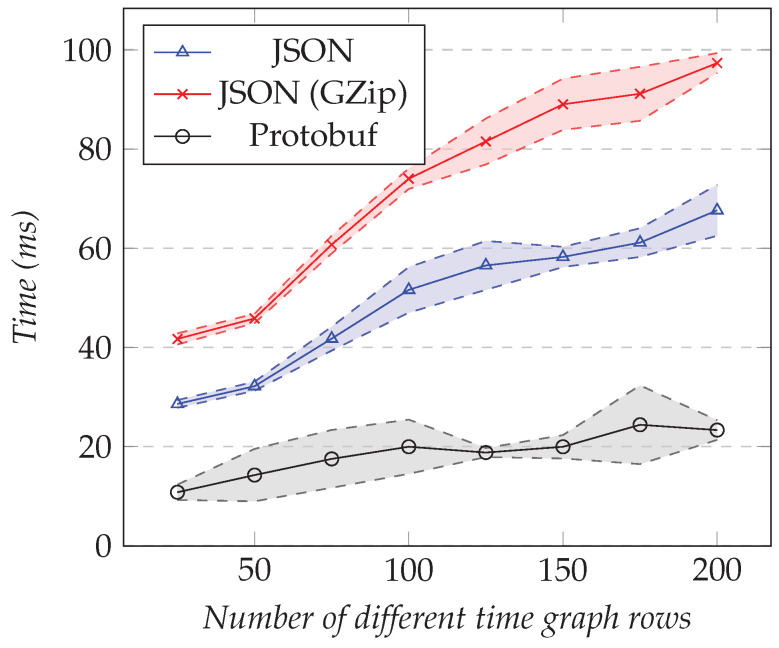
Comparison of the performance overhead for requesting the time graph row models. Fixed resolution set to 1000, changing the number of time graph rows.

We measured the execution time overhead for the same test configurations as the previous section. Figure 14 shows the results for the first test. Figure 15 shows the results for the second test.

As the resolution increased, the execution time overhead also increased, to exceed 130 ms for a resolution of 4000 points if using compression, while it was nearly 30 ms for Protobuf. Table 3 presents the execution time overhead as a percentage.

Compressed JSON led to the worst results, having a 13.4% overhead for a resolution of 500 and 11.2% for a resolution of 4000 points. Protobuf, on the other hand, scaled well, having 5.2% and 2.4% overhead, respectively. In between, we had 11.3% overhead for a resolution of 500 and 7.8% overhead for a resolution of 4000 by using JSON. The same observation as for the XY models holds: when we requested higher resolutions, the server spent more time computing the model than serializing it.

Akin to XY models, as the number of time graph rows increased, using compression led to the highest overhead for the execution time, reaching almost 100 ms for a resolution of 4000 points. On the other hand, Protobuf had the smallest overhead, with less than 25 ms. Finally, Table 4 shows the overhead as a percentage.

Using compression reached 13.4% overhead for 25 entries and 29.4% for 200 entries. Protobuf had the smallest cost, leading to 3.5% for 25 time graph rows and 6.8% for 200. In between, we had respectively 9.6% and 21.3% for JSON. Furthermore, as for the XY models, when querying more time graph rows from the Data Provider, the server spent more time serializing the model than computing it.

### 4.5. Scalability

In this section, we evaluate how our proposed solution scaled with the size of the trace. For each view model, we are interested in measuring how the size of the trace affects the data transferred and the execution time overhead per request. The traces used were also generated using LTTng. Their content in terms of the number of threads and number of events were not relevant for this test; we only took into consideration the size on disk.

#### 4.5.1. XY Models

The trace size should not have a significant impact on the amount of data transferred for XY models. In this test, we fixed the number of XY series to one and the resolution to one-thousand, and we changed the trace size. In theory, given that we always query for a constant number of points and a constant number of series, the size of the data transferred should be constant. However, as shown in Table 5, this was not necessarily the case for JSON and compressed JSON.

The reason why the amount of data transferred over Protobuf was constant is that each field of the model was serialized with a fixed number of bytes. The *y* property in the XY model is an array of double, and Protobuf constantly uses 8 B to encode a double value. The *x* property is an array of long, and Protobuf uses varints to encode them. Varints are a method for serializing an integer in one or more bytes depending on the value (leading null bytes are skipped). Since we used Unix Epoch times in nanoseconds in *x*, every value was large, and Protobuf constantly uses 8 B. However, we were able to obtain a size reduction by using differential time values, which are much smaller, as follows:

(4) $$\begin{array}{c} x[0]=\mathrm{Unix}\;\mathrm{Epoch}\;\mathrm{start}\;\mathrm{time}\;\mathrm{in}\;\mathrm{nanoseconds}\\ x[1]=\mathrm{Unix}\;\mathrm{Epoch}\;\mathrm{time}\;\mathrm{value}\;\mathrm{for}\;x[1]-x[0]\\ x[n]=\mathrm{Unix}\;\mathrm{Epoch}\;\mathrm{time}\;\mathrm{value}\;\mathrm{for}\;x[n]-x[n-1] \end{array}$$

For example, if we have originally *x* = [1000010, 1000020, 1000031, 1000041], we would transfer *x* = [1000010, 10, 11, 10]. This technique led to transferring fewer bytes. Indeed, when we sent all the Unix Epoch times directly, we would transfer 17.0 KB for Protobuf instead of 12.1 KB. Nonetheless, as shown by the results, the amount of data transferred over Protobuf was still constant. The results may change if we insert something else in *x*. For JSON, each field of the model was serialized into a string where each character was encoded with 1 B. Thus, the number of bytes needed to encode a long or a double value can vary. For compressed JSON, the size depended greatly on the data to be compressed. For example, if the *y* contains only 0 s, compression would be optimal. Finally, the differences in terms of data transferred were not significant compared to the difference between the trace sizes.

The same observation holds for the execution time overhead. Given that we always received a constant amount of data, the absolute execution time overhead in milliseconds should be roughly constant. What varies is the percentage overhead. Indeed, as the size of the trace increased, the time required to query the Data Provider increased as well, simply because the size of the state model was bigger. This led to a lower percentage overhead as the trace size increased. We ran the same test as previously; we fixed the number of XY series to one, fixed the resolution to one-thousand, and changed the trace size. Table 6 shows the results.

#### 4.5.2. Time Graphs Models

The size of the trace should affect the amount of data transferred. A trace of a few megabytes does not contain the same number of events as a trace of a few gigabytes. Thus, as the size of a trace increased, the number of states per time graph row increased as well, and this number influenced the size of the data transferred. In this test, we fixed the number of time graph rows to 25 and the resolution to 1000, and we changed the trace size. We compared the data transferred for each serialization format. Table 7 shows the results of the test.

We observed that the difference in the amount of data transferred was significant between the 1 MB trace and the 500 MB trace. From 500 to 2500 MB, the increase was slower and seemed to eventually reach a plateau. This can be explained by an optimization made by the Data Provider. If a state requires less than one pixel to be drawn, the Data Provider will not return it. We fixed the resolution to one-thousand, so in the worst-case scenario, we had one-thousand states, which is one state per pixel.

As the amount of data transferred increases while the size of the trace increases, the execution time overhead should also increase. As the data transferred, we should eventually reach a plateau. Table 8 shows the results of the execution time overhead. The same configuration as for the data transfer test was used. As expected, the results showed that a plateau was reached.

### 4.6. Storage

The client–server architecture that we proposed does not use additional disk storage. The view models are generated on-demand and are stored in RAM for caching, but are not written permanently on disk. For the local use case, everything works as before: the client and the server are running on the same machine, and the server stores the trace and the state model on disk. However, for the distributed use case, with the client and the server on different devices, this architecture guarantees that the client no longer needs to store the trace and the state model. Furthermore, the computer running the client does not require any specific software installation, except for a compatible modern browser (e.g., Chrome or Firefox).

### 4.7. Rendering Time

Once the model is received, the client needs to draw it. In this test, we evaluated how much time was required to draw the views, according to the view models, the number of XY series or time graph rows, and the resolution. Given that obtaining the model was independent of the drawing operation, the tests in this section used randomly generated models.

#### 4.7.1. XY Charts

**Figure 16:**
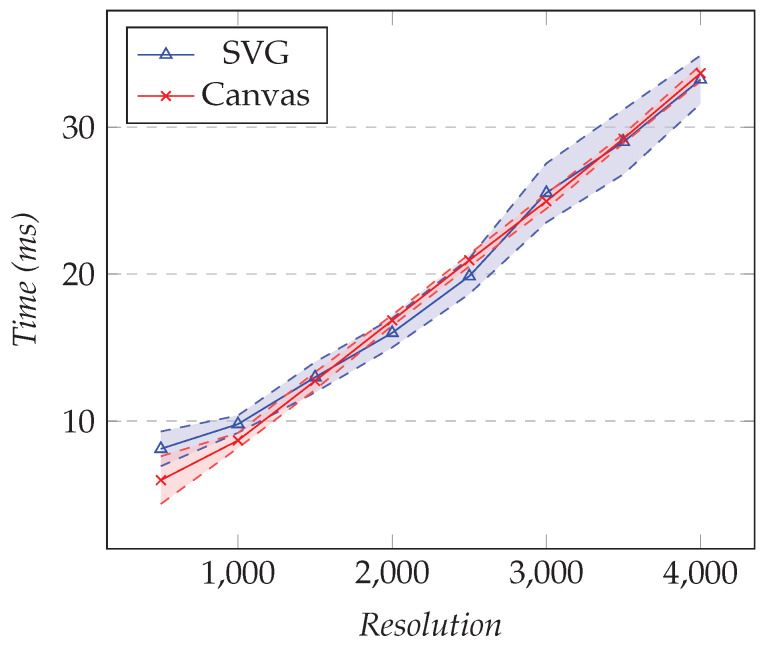
Comparison of the rendering time for an XY chart. The number of series is fixed to 1, and the resolution varies.

**Figure 17:**
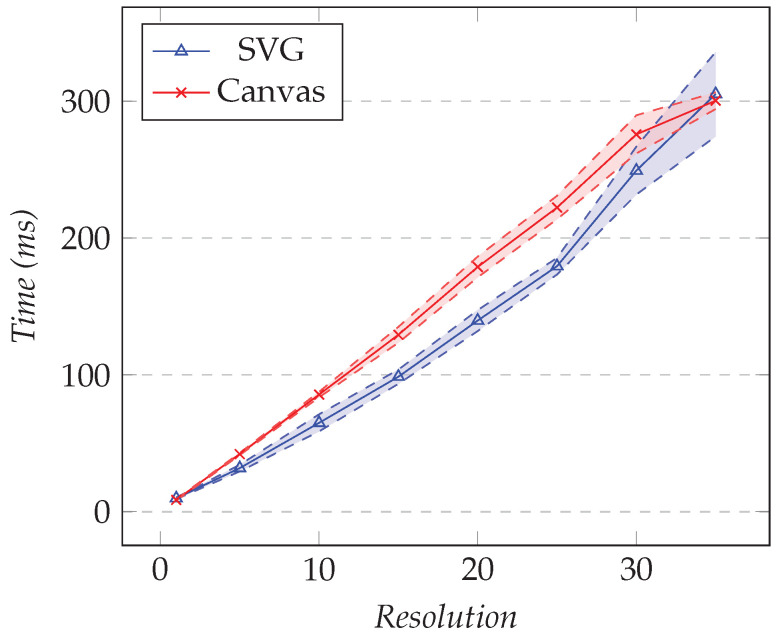
Comparison of the rendering time for an XY chart. The resolution is fixed to 1000, and the number of series varies.

Several open-source chart libraries are available. They either chose an SVG-based approach or a canvas-based approach. We used Chart.js for the canvas-based library and HighCharts for the SVG-based library. In this test, we compared the rendering time of both techniques. In the first test, we fixed the number of series to one and changed the resolution. In the second test, we fixed the resolution to 1000 and changed the number of XY series. Figure 16 shows the results for the first test and Figure 17 the results for the second.

As the number of points increased, the rendering times for SVG and canvas were very close. Less than 10 ms were needed to render 500 points and around 35 ms for 4000 points.

As the number of XY series increased, we saw that the rendering time for SVG was slightly better than canvas. Approximately 300 ms was needed to render the 35 XY series. However, in practice, a user would rarely show that many series on a chart.

#### 4.7.2. Time Graph Charts

We did not find a time graph chart library that supports nanoscale precision, so we implemented our time graph chart, which uses a canvas-based library. The chosen library, called PixiJS, uses hardware acceleration. In the first test, we fixed the number of time graph rows to 25. Given that we generated the model, each time graph row had the same number of states, and we changed this value for the first test. In the second test, we fixed the number of states to 1000, and we changed the number of time graph rows. Table 9 shows the results for the first test and Table 10 the results for the second.

### 4.8. Total Elapsed Time

Finally, in this section, we compare the total elapsed time for showing a view with our client–server solution and with the standalone approach. The elapsed time is the sum of the time to query the Data Provider and to show its results on a view. We used the 2.47 GB trace from the previous section and issued requests covering the whole time interval of the trace. We requested the XY model and the time graph model. The following is a description of the two configurations studied:

- Standalone: locally in Trace Compass, with the implementation of the Data Provider. The Java-based views call the Data Provider explicitly and display the results;
- Client-server: locally in TraceScape, with Trace Compass as a trace analysis server and the Protobuf serialization format. The JavaScript-based views make HTTP requests to the server and display the results.

#### 4.8.1. XY Models

**Figure 18:**
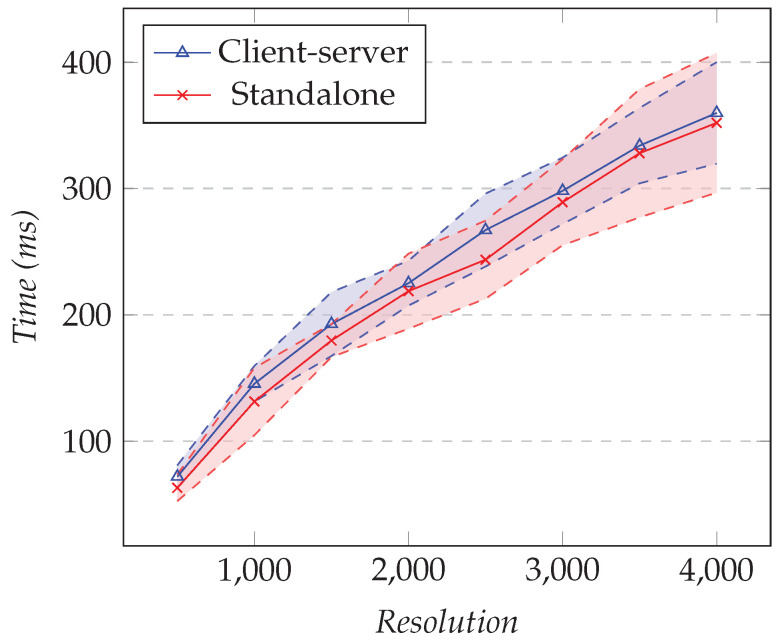
Comparison of the total elapsed time for the XY model. The number of series is fixed to 1, and the resolution varies.

**Figure 19:**
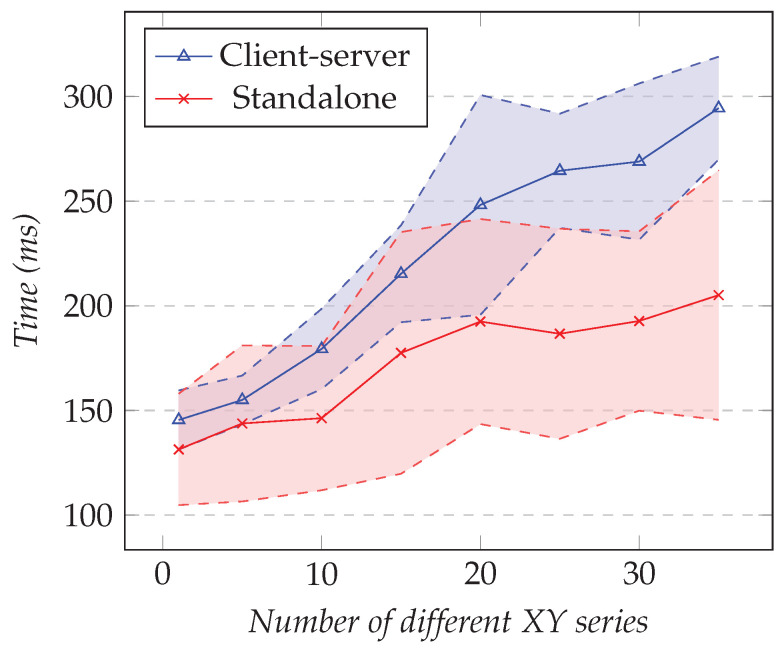
Comparison of the total elapsed time for the XY model. Fixed resolution set to 1000 and changing the number of series.

In the first test, we fixed the number of XY series to one and changed the resolution. In the second test, we fixed the resolution to 1000 and changed the number of different XY series. Figure 18 shows the results of the first test and Figure 19 the results for the second test.

As the resolution increased, the difference in overhead between the client–server approach and the standalone approach was not significant. From the results, the maximum cost was around 25 ms and the minimum was about 6 ms.

However, as the number of XY series increased, the difference in overhead between our solution and the standalone approach was noticeable. This can be explained by the fact that the XY chart library used is SVG-based. For each additional series, 1000 elements were appended in the Document Object Model (DOM), and this impacted the performance significantly. For 35 XY series, the difference was around 90 ms.

#### 4.8.2. Time Graph Models

**Figure 20:**
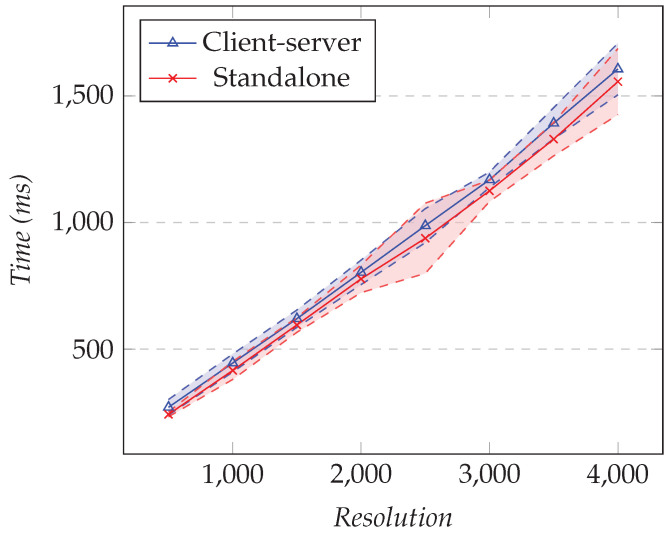
Comparison of the total elapsed time for the time graph row model. The number of time graph rows is 25, and the resolution varies.

**Figure 21:**
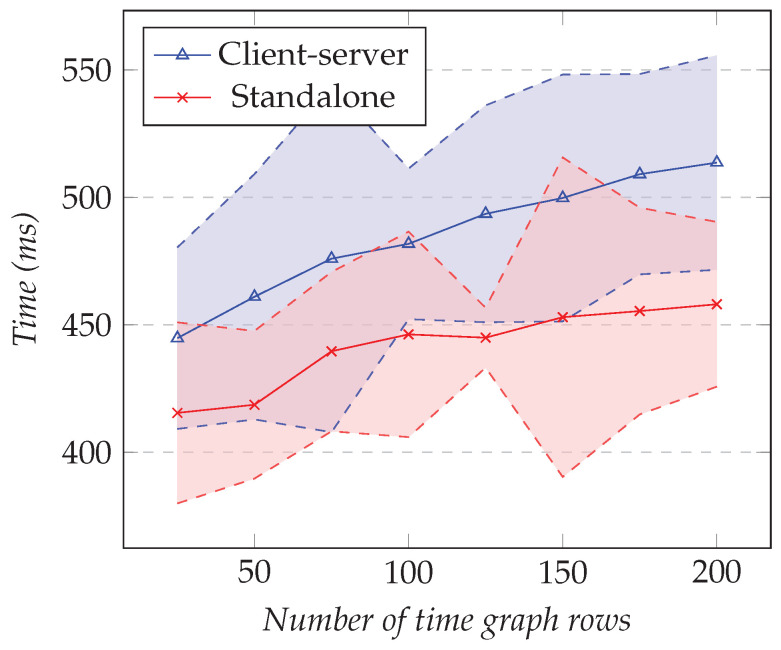
Comparison of the total elapsed time for the time graph row model. The resolution is fixed to 1000, and the number of time graph rows varies.

In the first test, we fixed the number of time graph rows to 25 and changed the resolution. In the second test, we fixed the resolution to 1000 and changed the number of different time graph rows. Figure 20 shows the results of the first test and Figure 21 the results for the second test.

As the resolution increased, the elapsed time for our solution and the standalone approach was close. From the results, the overhead was between 25 ms and 65 ms, but those values are relatively small compared to the total elapsed time.

However, as the number of time graph rows increased, the difference in elapsed time between the client–server approach and the standalone approach grew slightly. From the results, the overhead was between 30 ms and 55 ms. Unlike the results in the initial test, those values were noticeable compared to the total elapsed time.

## 5. Discussion

In Section 4, we showed that our solution scales well for large traces, both in terms of data transferred and execution time overhead. While compressed JSON and Protobuf were close when it comes to sending the smallest amount of data over the network, Protobuf had the lowest execution time overhead. Depending on the model requested, the resolution, and the number of XY series or time graph rows, using the worst case for Protobuf required less than 700 KB and less than 50 ms overhead per request for a trace of 2.47 GB. In comparison with the standalone approach, and as per our criteria from Section 1, our proposed solution adds a small and acceptable cost to the total elapsed time.

However, our solution is not optimal for small traces with the size on disk of only a few megabytes. Indeed, we could send more data over the network than the trace size. Moreover, we do not know how many requests will be made by the client during a session. For example, with a 1 GB trace, if each request leads to the worst-case scenario where we transfer 700 KB per request and if the client makes more than 1430 requests, we will transfer in total the size of the trace. In practice, this should not happen often. Moreover, losing some time on small traces should not be a concern, as they require very little time to process anyway.

Other communication protocols may be as good as or better than HTTP. We investigated RPC-based protocols such as JSON-RPC and gRPC. We excluded the first because we wanted to minimize the amount of data transferred. The second protocol, on the other hand, uses Protobuf as a serialization format and runs over the HTTP/2 protocol [32]. However, browsers do not fully support the gRPC protocol, and we needed a particular configuration involving a reverse proxy to make it work with the trace analysis server. Hopefully, this limitation will be solved in the future, so we can evaluate precisely how our REST approach compares to an RPC approach.

We proved that our solution is efficient, but we also said that it is flexible, maintainable, and reusable. One of our focuses in this paper, specifically during the Evaluation section, was to measure the performance, but it is more difficult to measure and assess those qualities precisely. Because we applied design patterns and software architecture principles, we can intuitively say that our solution has those qualities. However, this intuition is profoundly influenced by the developer’s work experience. This aspect is another limitation of our work.

## 6. Conclusions and Future Work

In this paper, we presented a client–server architecture for large trace analysis and visualization, which can integrate well with an IDE. By applying the separation of concerns principles, each component of the architecture has a specific role and responsibility, making them reusable and independently maintainable. We introduced the Trace Analysis Server Protocol (TASP) and implemented the API Gateway and the server-side service discovery pattern to ensure that the architecture is flexible and supports multiple heterogeneous clients.

On the server-side, instead of sending the state models, we introduced three new architectural components in the trace analysis process to reduce the amount of data transferred. On the client-side, we presented some techniques to avoid communication between the client and the server and to optimize the requests to limit the amount of data transferred. To evaluate our solution, we implemented the server architecture in the Trace Compass project and implemented a JavaScript web client. We measured the data transferred per request according to the serialization format, the view model, the number of entries, and the resolution. We found that compressed JSON (GZip) and Protobuf were close when it comes to sending the smallest amount of data. However, when we compared the execution time overhead, Protobuf was significantly better than compressed JSON. Then, we showed that our solution scaled with large traces. We also discussed why it was not optimal for small traces. Finally, in comparison with the standalone approach, our solution added a small and acceptable overhead for the total elapsed time.

We believe that our proposed architecture would lead to better integration of tracing and trace analysis in IDEs to better support developers. To our knowledge, this is the first trace visualization tool that addresses the challenges brought forward by the recent modularization of IDEs, with a frontend user interface and backend servers for language parsing, debugging, and now tracing. Our solution is well suited for IoT systems, where resource constraints must be considered. As the systems are becoming more and more complex, we must adapt our tools to detect defects quickly and effectively. Future work could investigate how IDEs could use some components from our architecture and identify for the developer which part of the code causes problems, using the trace analysis results.

## Figures and Tables

**Table 1:** Relativeexecution overhead for requesting the XY model for 1 series.

	Overhead (%)		
**Resolution**	**JSON**	**JSON (GZip)**	**Protobuf**
500	7.1	11.4	9.9
1000	5.2	7.8	4.8
1500	4.6	6.7	3.2
2000	3.8	6.6	2.7
2500	3.8	6.5	2.7
3000	3.7	6.2	2.0
3500	3.7	6.5	1.9
4000	3.4	7.3	1.8

**Table 2:** Relative execution overhead for requesting the XY model. Fixed resolution set to 1000.

	Overhead (%)		
**Number of XY Series**	**JSON**	**JSON (GZip)**	**Protobuf**
1	5.1	7.8	4.8
5	9.2	12.8	5.5
10	13.0	21.2	7.0
15	18.5	25.1	8.6
20	25.3	34.3	9.2
25	29.7	28.4	10.8
30	36.2	47.3	11.7
35	40.9	48.0	13.2

**Table 3:** Relative execution overhead for requesting the time graph row models. Fixed number of time graph rows set to 25.

	Overhead (%)		
**Resolution**	**JSON**	**JSON (GZip)**	**Protobuf**
500	11.3	13.4	5.2
1000	9.6	13.3	3.5
1500	9.6	13.5	3.0
2000	9.5	12.5	4.2
2500	8.9	12.1	2.9
3000	8.0	11.4	3.0
3500	7.6	11.3	3.3
4000	7.8	11.2	2.4

**Table 4:** Relative execution overhead for requesting the time graph row models. Fixed resolution set to 1000.

	Overhead (%)		
**Time Graph Row Count**	**JSON**	**JSON (GZip)**	**Protobuf**
25	9.6	13.4	3.5
50	10.6	14.7	4.6
75	13.8	19.8	5.6
100	16.7	23.7	6.4
125	18.2	25.6	6.0
150	18.5	27.8	6.2
175	19.3	27.7	7.4
200	21.3	29.4	6.8

**Table 5:** Data transferred for requesting the XY model according to the trace size.

	Data Transferred (KB)		
**Trace Size (MB)**	**JSON**	**JSON (GZip)**	**Protobuf**
1.1	40.1	1.3	12.1
57.8	44.5	1.6	12.1
124.2	43.6	2.4	12.1
471.8	49.4	8.3	12.1
1507	49.2	10.3	12.1
1965	51.4	9.0	12.1
2470	51.4	10.1	12.1
3381	51.4	9.7	12.1

**Table 6:** Absolute execution time overhead for requesting the XY model according to the trace size.

	Execution Time (ms)		
**Trace Size (MB)**	**JSON**	**JSON (GZip)**	**Protobuf**
1.1	5.25	5.8	5.34
57.8	5.11	6.3	4.14
124.2	4.76	5.74	5.16
471.8	4.83	6.24	4.75
1507	5.3	6.65	4.91
1965	4.86	6.41	5.05
2470	5.18	6.68	4.55
3381	5.08	7.31	6.09

**Table 7:** Data transferred for requesting the time graph row model according to the trace size.

	Data Transferred (KB)		
**Trace Size (MB)**	**JSON**	**JSON (GZip)**	**Protobuf**
1.1	143	11.5	21.3
57.8	215	22.6	32.7
124.2	862	87.9	126
471.8	1100	122	170
1507	1100	120	167
1965	1200	131	181
2470	1200	131	187
3381	1300	139	189

**Table 8:** Absolute execution time overhead for requesting the time graph row model according to the trace size.

	Execution Time (ms)		
**Trace Size (MB)**	**JSON**	**JSON (GZip)**	**Protobuf**
1.1	6.34	7.76	6.16
57.8	7.54	10.0	7.07
124.2	20.27	29.03	9.80
471.8	26.30	38.73	10.31
1507	25.66	38.11	11.01
1965	28.72	39.58	10.86
2470	27.81	40.34	12.10
3381	29.22	42.35	11.03

**Table 9:** Rendering time for the time graph chart with randomly generated models. The number of time graph rows is fixed to 25.

Resolution (Number of States)	Rendering Time (ms)
500	6.48
1000	6.13
1500	8.09
2000	13.55
2500	18.70
3000	23.59
3500	29.00
4000	34.85

**Table 10:** Rendering time of the time graph chart with randomly generated models. Fixed resolution set to 1000.

Number of Time Graph Row	Rendering Time (ms)
25	6.79
50	14.27
75	25.18
100	33.85
125	39.81
150	49.76
175	62.70
200	68.67

## Data Availability

All source codes and trace data are available as an open-source project in the following GitHub repository: https://github.com/cheninator/trace-scape (accessed on 10 February 2021).
